# Contesting the dogma of an age-related heat shock response impairment: implications for cardiac-specific age-related disorders

**DOI:** 10.1093/hmg/ddu073

**Published:** 2014-02-19

**Authors:** Alisia Carnemolla, John P. Labbadia, Hayley Lazell, Andreas Neueder, Saliha Moussaoui, Gillian P. Bates

**Affiliations:** 1 Department Medical and Molecular Genetics, King's College London, 8th Floor Tower Wing, Guy's Hosptial, Great Maze Pond, London SE1 9RT, UK; 2 Novartis Institute for Biomedical Research, Neuroscience Discovery, Basel CH-4002, Switzerland

## Abstract

Ageing is associated with the reduced performance of physiological processes and has been proposed as a major risk factor for disease. An age-related decline in stress response pathways has been widely documented in lower organisms. In particular, the heat shock response (HSR) becomes severely compromised with age in *Caenorhabditis elegans*. However, a comprehensive analysis of the consequences of ageing on the HSR in higher organisms has not been documented. We used both HS and inhibition of HSP90 to induce the HSR in wild-type mice at 3 and 22 months of age to investigate the extent to which different brain regions, and peripheral tissues can sustain HSF1 activity and HS protein (HSP) expression with age. Using chromatin immunoprecipitation, quantitative reverse transcription polymerase chain reaction, western blotting and enzyme linked immunosorbent assay (ELISA), we were unable to detect a difference in the level or kinetics of HSP expression between young and old mice in all brain regions. In contrast, we did observe an age-related reduction in chaperone levels and HSR-related proteins in the heart. This could result in a decrease in the protein folding capacity of old hearts with implications for age-related cardiac disorders.

## INTRODUCTION

Ageing is defined as the physiological time-dependent decline in all biological processes arising from the accumulation of cellular damage (1,2). The function and plasticity of specific cells relies on a distinctive proteome which is constantly challenged by intrinsic and environmental stresses. Protein homeostasis (proteostasis) is critical for maintaining organismal viability and cell function. Protection against proteotoxicity involves four main stress response pathways: the heat shock response (HSR) (3), the unfolded protein response in the endoplasmic reticulum (UPR^ER^) (4), the unfolded protein response in the mitochondria (UPR^mt^) (5) and the oxidative stress response (OxSR) (6). These stress pathways together with the ubiquitin proteasome system and autophagy (for the clearance of irreversibly damaged proteins) represent the proteostasis network (7). An age-related decline in stress response pathways and the proteostasis network has been widely documented in lower organisms (8–10). In particular, the HSR becomes severely compromised with age in *Caenorhabditis elegans* (8). In mammals, the reduced ability of HS factor-1 (HSF1) to bind DNA and induce HSP70 up-regulation in liver and heart samples of old rats and mice (11–16), as well as the inability of the human brain to deal with chronic proteotoxic stress, such as in Alzheimer's disease, Parkinson's disease and Huntington's disease, has led to the assumption that the HSR is compromised as a consequence of ageing. However, an extensive and in-depth analysis of the effects of ageing in higher level organisms has not been documented.

In order to investigate the extent to which different brain regions and peripheral tissues can support HSF1 activity and the expression of HS proteins (HSPs), we used both HS and the inhibition of HSP90 (using NVP-HSP990) (17) to induce the HSR in wild-type (C57BL/6 X CBA)F1 mice at 3 and 22 months of age. Using chromatin immunoprecipitation (ChIP), quantitative reverse transcription polymerase chain reaction, western blotting and enzyme linked immunosorbent assay (ELISA), we were unable to detect a difference in the level or kinetics of HSP expression between young and old mice in all brain regions. We did observe an age-related reduction in the basal levels of HSPs and other HSR-related proteins in the heart, and in this tissue, the fold induction of HSP70 and HSP25 upon HSP990 treatment was reduced at the protein but not the RNA level. This would be expected to result in a decrease in the protein folding capacity of old hearts with implications for age-related cardiac disorders. We also describe a heart-specific compensatory mechanism for HSF1 regulation. Therefore, in contrast to the situation in lower organisms, our data do not support a tissue wide age-related decline in the HSR in mammals.

## RESULTS

### HSP induction is not impaired in old mice upon HS

There is considerable evidence to suggest that the HSR may be impaired in higher organisms as a consequence of ageing (11–16). To study the effect of ageing on the HSR, we challenged mice with an HS to determine the extent to which the proteostasis network was able to respond to a highly damaged cellular environment arising from a heat stress. Wild-type (C57BL/6 X CBA)F1 mice at 3 and 22 months of age were heat shocked for 15 min at 41.5°C ± 0.2°C using a heating pad and tissues were harvested 4 h later. The expression levels of the major HS genes were assessed by RT–qPCR and western blotting. We did not detect any differences in the thermal responses between young and old animals as in both cases, the body temperature rose from 36.5°C ± 0.2°C to 41.5°C ± 0.2°C over the course of 6–7 min. Therefore, 3- and 22-month-old mice were exposed to a similar thermal stress during HS.

**Figure 1.**
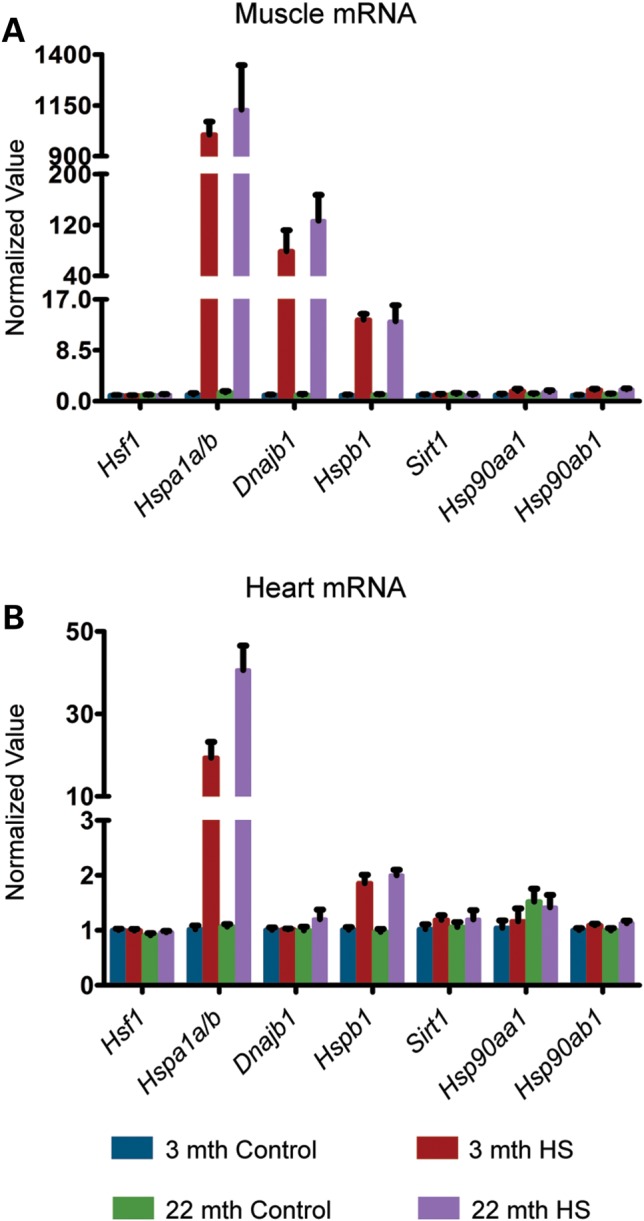
Effect of HS on HSP mRNA induction in young and old mice. Tissues were taken from 3- and 22-month-old mice 4 h after HS (15 min at 41.5°C). Controls were maintained at 36.9°C during this period. (**A**) qPCR analysis of the expression levels of *Hsf1*, *Hspa1a/b*, *Dnajb1*, *Hspb1*, *Sirt1*, *Hsp90aa1* and *Hsp90ab1* in the muscle. (**B**) qPCR analysis of the expression levels of *Hsf1*, *Hspa1a/b*, *Dnajb1*, *Hspb1*, *Sirt1*, *Hsp90aa1* and *Hsp90ab1* in the heart. Values were calculated relative to vehicle-treated young mice. Data are mean ± SEM. 4<*n*< 6/group. mth, months.

Under these conditions, the brain does not experience an HS (see Materials and Methods) limiting our analysis to peripheral tissues such as muscle (quadriceps) and heart. It is interesting to note that the pattern of HS gene up-regulation was different between these two tissues. *Hspa1a/b* (HSP70), *Dnajb1* (HSP40) and *Hspb1* (HSP25) were highly up-regulated in muscle (Fig. 1A), whereas in the heart, only *Hspa1a/b* was robustly induced (Fig. 1B). This tissue specificity was also observed for the two *Hsp90* isoforms: both were moderately up-regulated (1.5–2-fold) in the muscle but not in the heart (Fig. 1A and B). We found that despite this challenge to the proteostasis network, at the RNA level, there was no difference in the extent of induction of these three chaperones between young and old mice in both tissues. Consistent with previous data showing that the induction of the HSR does not elicit an up-regulation of HSF1 (18), the expression of *Hsf1* mRNA was not modulated by heat stress in both tissues (Fig. 1). We also studied the effect of hyperthermia on *Sirt1*, a known positive regulator of HSF1 (19), and found that, similar to *Hsf1*, the expression of this sirtuin was not affected by HS in the muscle or heart, both in young and old mice (Fig. 1).

To investigate whether the pattern of mRNA up-regulation was reflected at the protein level, we measured the relative levels of HS proteins. Despite the fact that at 4 h after HS, HSP induction is just beginning, it was possible to detect a pronounced induction for HSP70, the levels of which were not significantly different between young and old mice (Supplementary Material, Fig. S1A and B). Consistent with the mRNA data, there was no variation at the protein level for both HSF1 and SIRT1 4 h after HS (Supplementary Material, Fig. S1A and B), when HSF1 had already reverted to its inactive, non-hypershifted state in heat-shocked samples (Supplementary Material, Fig. S1B). Taken together, these results demonstrate that hyperthermia can induce the HSR to the same extent in old as in young mice.

### The HSR is not impaired in old mice when treated with an HSP90 inhibitor

The inability of the brain to respond to hyperthermia led us to explore alternative approaches to study the HSR in the CNS of mice. The HSR can be induced by targeting the ATP pocket of HSP90 (20–22), and we have previously shown that NVP-HSP990 (referred to herein as HSP990) (23,24) is a very strong brain penetrant HSP90 inhibitor that can be used for this purpose (17).

**Figure 2.**
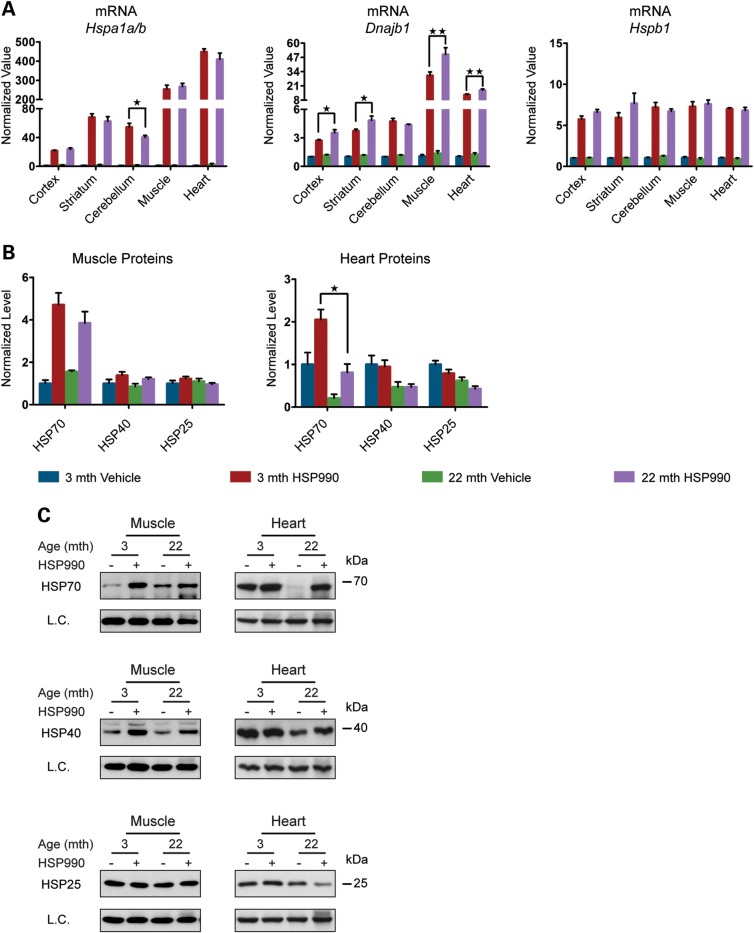
HSP induction is not impaired in old mice. Tissues were taken from 3- and 22-month-old mice 2 h after treatment with HSP990 (12 mg/kg) or vehicle. (**A**) qPCR analysis of the expression levels of *Hspa1a/b*, *Dnajb1* and *Hspb1* in brain regions and peripheral tissues. Values were calculated relative to vehicle-treated young mice. (**B**) The relative protein level of HSP70, HSP40 and HSP25 in the muscle and heart. Densitometric values were calculated relative to vehicle-treated young mice. (**C**) Representative western blots used for the quantification in (B). Data are the mean ± SEM. 4<*n* < 8/group. *P < 0.05; **P < 0.01: asterisk indicates the statistically significant difference in the level of induction; Muscle, quadriceps; L.C., loading control; mth, months.

To study the effect of ageing on the HSR in different mouse brain regions (cortex, striatum, cerebellum, hippocampus, brainstem and the ‘rest of brain’) and peripheral tissues [muscle (quadriceps), heart and liver], young and old mice at 3 and 22 months of age were treated with a single acute oral dose of HSP990 (12 mg/kg) and the expression levels of the major HS genes were assessed 2 h after treatment by RT–qPCR and western blotting. At this time point, we would expect mRNA levels to be highly induced (17) and we found all three chaperone genes (*Hspa1a/b*, *Dnajb1* and *Hspb1*) to be up-regulated in both young and old mice in all tissues analysed (Fig. 2A and Supplementary Material, Fig. S2A). Contrary to expectations, we did not detect significant differences in the induction of *Hspb1* mRNA levels between young and old mice (Fig. 2A and Supplementary Material, Fig. S2A) and the levels of *Dnajb1* were significantly higher in nearly all of the 22-month tissues when compared with those from mice at 3 months of age (Fig. 2A and Supplementary Material, Fig. S2A). *Hspa1a/b* induction was comparable between young and old mice in the cortex, striatum, brainstem, rest of brain, muscle and heart; however, induction of *Hspa1a/b* at this time point was lower in old when compared with young mice in the cerebellum (23%), hippocampus (32%) and liver (43%), although old animals were still capable of potently up-regulating *Hspa1a/b* (Fig. 2A and Supplementary Material, Fig. S2A). At the protein level, at 2 h post-dosing, HSP induction is in the very early stages. HSP70 was the only chaperone for which there was any indication of up-regulation in the brain, and even if this was statistically significant, the fold change was too small to be able to draw any comparative conclusions. In the periphery, the pattern of chaperone up-regulation and the degree of induction were tissue-specific. HSP70 was still the most highly induced chaperone, the level of which was comparable in muscle between young and old mice, but was significantly lower in the heart and liver of old mice (Fig. 2B and C and Supplementary Material, Fig. S2B and C). Taken together, these results suggest that the ability to up-regulate HSPs is not lost in the brains of old mice, but some peripheral tissues do show age-related alteration in the regulation of chaperone levels.

### HSF1 activation and DNA-binding capacity are not negatively affected by ageing

**Figure 3.**
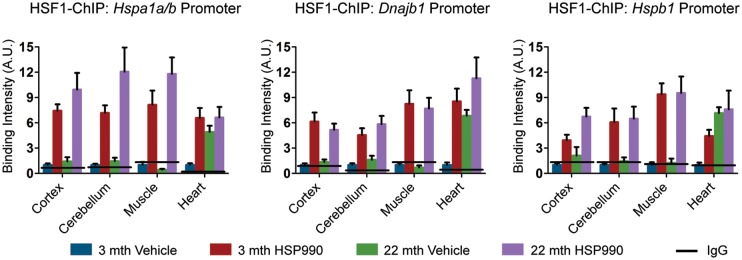
HSF1 DNA-binding capacity is not negatively affected by ageing. Tissues were taken from 3- and 22-month-old mice 2 h after treatment with HSP990 (12 mg/kg) or vehicle. Levels of HSF1 bound to HS promoters as determined by ChIP. Solid line represents the IgG control values. Data are the mean ± SEM. 4<*n* < 8/group. Muscle, quadriceps; mth, months; A.U., arbitrary units.

**Figure 4.**
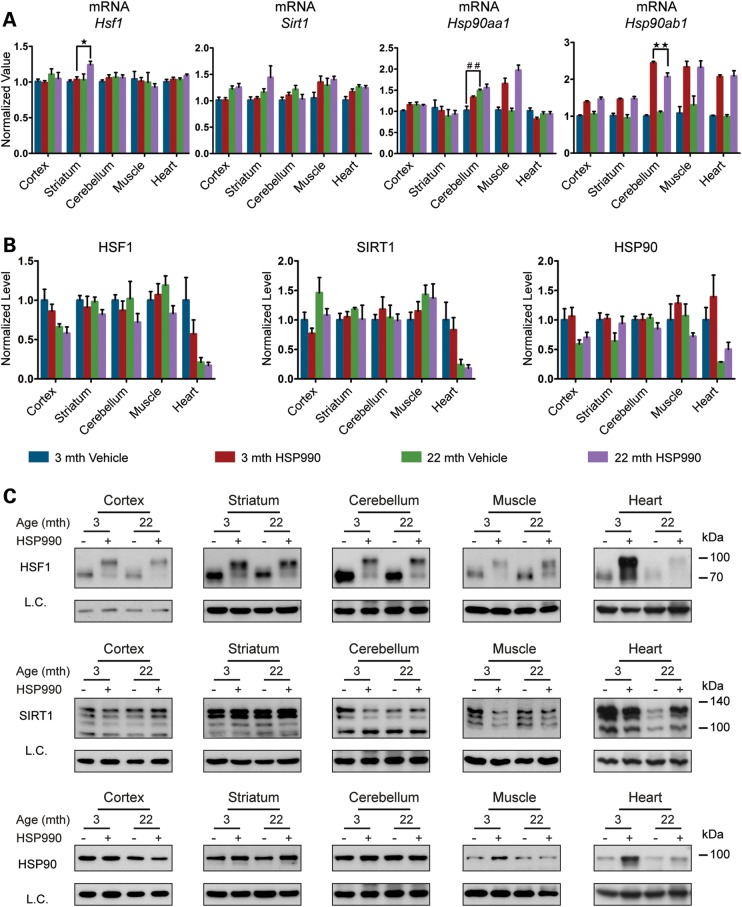
Analysis of HSR regulatory proteins. Tissues were taken from 3- and 22-month-old mice 2 h after treatment with HSP990 (12 mg/kg) or vehicle. (**A**) qPCR analysis of the expression levels of *Hsf1*, *Sirt1*, *Hsp90aa1* and *Hsp90ab1* in brain regions and peripheral tissues. Values were calculated relative to vehicle-treated young mice. (**B**) Relative protein level of HSF1, SIRT1 and HSP90 in brain regions and peripheral tissues. Densitometric values were calculated relative to vehicle-treated young mice. (**C**) Representative western blots for HSF1, SIRT1 and HSP90. Data are the mean ± SEM. 4<*n* < 8/group. *P < 0.05; **P < 0.01. Asterisk indicates the statistically significant difference in the level of induction; ^##^*P* < 0.01: Number sign indicates the statistically significant difference between vehicle groups. Muscle, quadriceps; L.C., loading control; mth, months.

The HS transcription factor HSF1 is the master regulator of the HSR and is essential for HSP induction in response to thermal stress *in vivo* (25). In response to protein misfolding, HSF1 is released from an inhibitory complex containing HSP90 (26–28), becomes hyperphosphorylated and translocates to the nucleus where it binds to the promoter regions of HSP genes to induce transcription (3). To determine whether this mechanism remains functional in old mice, a step by step analysis of HSF1 activation was conducted in tissues taken from mice 2 h post-dosing with HSP990 (12 mg/kg). Co-immunoprecipitation experiments revealed that HSF1 interacted with HSP90 in cortical samples from both young and old vehicle-treated mice (Supplementary Material, Fig. S3A) and consistent with the mechanism of HSF1 activation, 2 h after HSP990 treatment, HSP90 no longer co-immunoprecipitated with HSF1 in mice of both ages, indicating that HSF1 had dissociated from the inhibitory HSP90 complex (Supplementary Material, Fig. S3A). Western blotting revealed that HSF1 was hyperphosphorylated 2 h after HSP990 treatment in both young and old mice for all tissues analysed (Fig. 4C, Supplementary Material, Fig. S3A and S4C). We then monitored HSF1 DNA binding at the promoter region of the three main HSP genes: *hspa1a/b*, *Dnajb1* and *Hspb1*, in two different brain regions (cortex and cerebellum) and three peripheral tissues (muscle, heart and liver) by ChIP. We found HSF1 to be strongly bound to the promoters of all three HSP genes upon HSP990 treatment and contrary to previous data (11,29,30), we did not detect an age-dependent reduction in HSF1-binding (Fig. 3 and Supplementary Material, Fig. S3B). Therefore, we have no evidence to suggest that the dynamics of HSF1 activation were altered in old mice.

### HSR regulatory proteins are reduced in aged hearts

There was no difference between HSP990-treated young and old mice in the level of HSF1 binding to HSP gene promoters after HSR induction in all tissues examined (Fig. 3 and Supplementary Material, Fig. S3B). However, a marked increase in HSF1 binding to all three HS gene promoters was detected in the heart when comparing vehicle-treated old and young mice (Fig. 3). To elucidate the molecular mechanism responsible for this observation, we analysed HSF1 expression levels by RT–qPCR and western blotting. The levels of *Hsf*1 mRNA did not differ between vehicle-treated young and old mice for any tissues, including the heart, and did not increase upon HSR induction (Fig. 4A and Supplementary Material, Fig. S4A). The western analysis revealed that in the heart, but not in any other tissue, the level of HSF1 was dramatically lower in vehicle-treated old mice when compared with young mice (Fig. 4B and C and Supplementary Material, Fig. S4B and C), and despite these very low levels, HSP990 treatment did not result in HSF1 up-regulation (Fig. 4B and C). The retention of HSF1 at the promoters of HSP genes in the heart might therefore be a compensatory mechanism for the reduced availability of this transcription factor.

We next monitored the expression levels of SIRT1 and HSP90 as known positive and negative regulators of HSF1 (19,26–28). At the mRNA level we did not detect any variation in *Sirt1* mRNA upon HSP990 treatment in either young or old mice (Fig. 4A and Supplementary Material, Fig. S4A). A consistent, although mild (∼2-fold or lower), up-regulation of *Hsp90ab1* (constitutive isoform) was detected in all tissues at both ages (Fig. 4A and Supplementary Material, Fig. S4A). The inducible *Hsp90aa1* isoform was only found to be very moderately (2-fold or less) up-regulated in muscle 2 h after HSP990 treatment irrespective of age (Fig. 4A and Supplementary Material, Fig. S4A). At the protein level, we did not detect a significant difference between young and old mice in the total amount of either SIRT1 or HSP90, although heart tissue showed an age-related decline in both SIRT1 and HSP90 protein, as had been observed for HSF1 (Fig. 4B and C and Supplementary Material, Fig. S4B and C).

### The dynamics of HSP induction are not altered in aged mice

**Figure 5.**
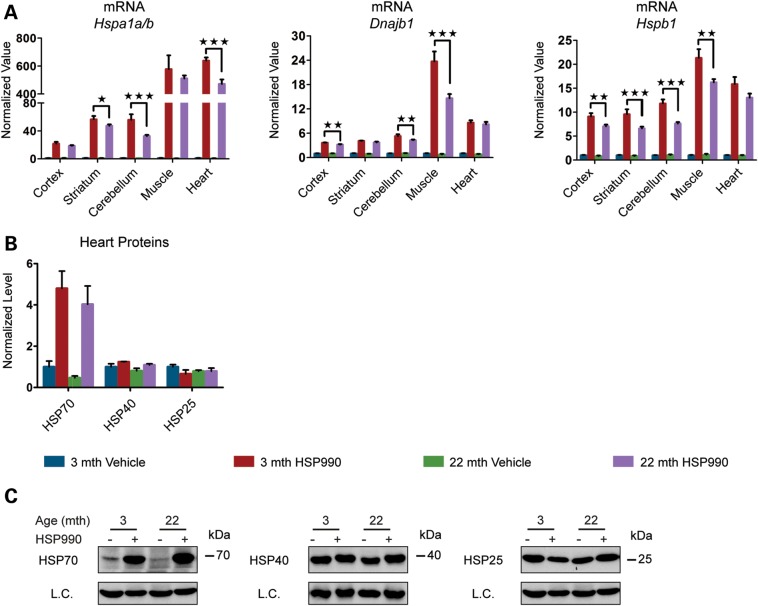
Analysis of the dynamics of HSP induction. Tissues were taken from 3- and 22-month-old mice 4 h after treatment with HSP990 (12 mg/kg) or vehicle. (**A**) qPCR analysis of the expression levels of *Hspa1a/b*, *Dnajb1* and *Hspb1* in brain regions and peripheral tissues. Values were calculated relative to vehicle-treated young mice. (**B**) Relative protein level of HSP70, HSP40 and HSP25 in the heart. Densitometric values were calculated relative to vehicle-treated young mice. (**C**) Representative western blots for HSP70, HSP40 and HSP25. Data are the mean ± SEM. 3 < *n* < 6/group. *P < 0.05; **P < 0.01; ***P < 0.001. Asterisk indicates the statistically significant difference in the level of induction. L.C., loading control; mth, months.

We have previously shown that the mRNA levels of *Hspa1a/b*, *Dnajb1* and *Hspb1* peak at ∼4 h post-dosing with HSP990 (17). To compare the dynamics of HSP gene expression, young and old mice were sacrificed 4 h after an acute dose of vehicle or HSP990 (12 mg/kg) and mRNA levels monitored by RT–qPCR. As for 2 h post HSP990 treatment, all three HSP genes were highly induced in all brain regions and peripheral tissues in both young and old mice (Fig. 5A and Supplementary Material, Fig. S5A). Interestingly, in the brain and liver, the mRNA levels of all three HSPs were similar to those that had been detected at 2 h post-dosing, suggesting that HSP mRNA synthesis had been repressed. It is well established that the expression of the HSP genes is auto-regulated (31). As such, it is possible that the lower HSP mRNA levels detected at 4 h post-dosing in old when compared with young HSP990-treated mice indicate that HSP gene expression had peaked and consequently had become repressed, slightly earlier in the old mice. Once again, the pattern of induction in the muscle and heart was chaperone-specific (Fig. 5A and Supplementary Material, Fig. S5A). *Hspa1a/b* and *Hspb1* mRNA levels were higher at 4 h post-dosing, whereas *Dnajb1* levels were already lower than at 2 h post-dosing in both tissues. Comparison of the mRNA levels between young and old mice would suggest that where differences exist, they may reflect a more rapid attenuation of HS gene expression in old mice (Fig. 5A and Supplementary Material, Fig. S5A).

In order to confirm this auto-regulatory process of mRNA expression by protein levels (31), we monitored HSP levels in the heart by western blotting. Interestingly, HSP70 levels were higher than at the 2 h time point and absolutely equivalent between young and old mice, suggesting that the difference detected at 2 h after HSP990 dosing had been redressed in old mice (Fig. 5B and C). As had been the case for the HS experiment, there was no significant up-regulation of both HSP40 and HSP25, suggesting that these chaperones may follow a different kinetics of accumulation (Fig. 5B and C).

**Figure 6.**
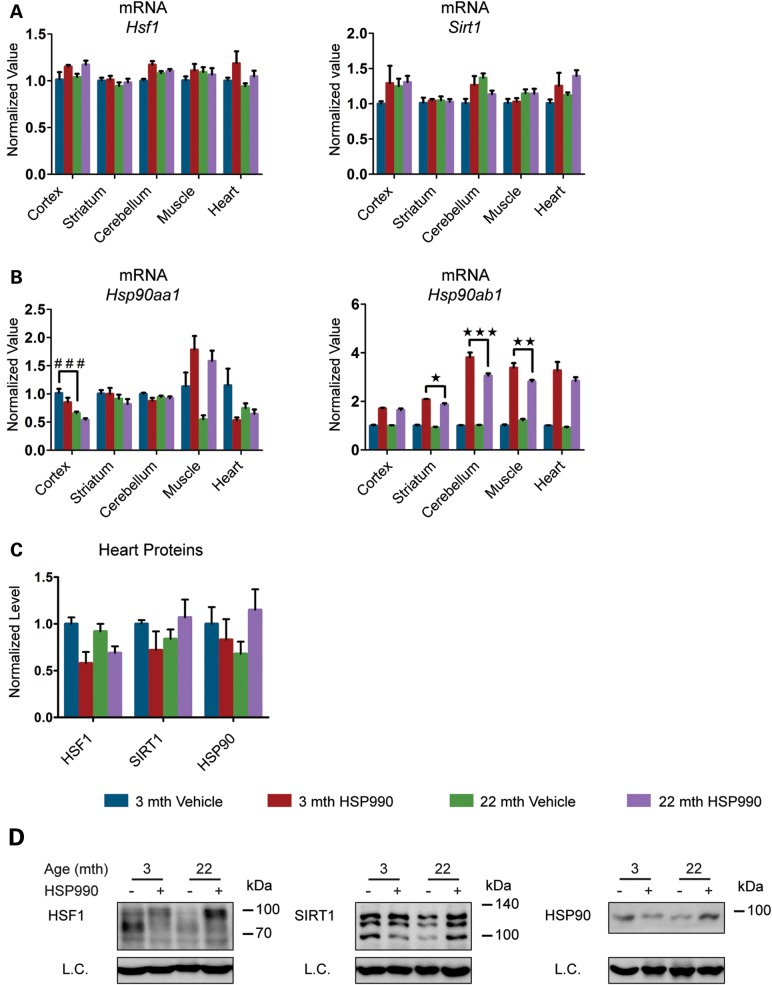
Analysis of the dynamics of HSR-regulatory proteins. Tissues were taken from 3- and 22-month-old mice 4 h after treatment with HSP990 (12 mg/kg) or vehicle. (**A**) qPCR analysis of the expression levels of *Hsf1* and *Sirt1* in brain regions and peripheral tissues. Values were calculated relative to vehicle-treated young mice. (**B**) qPCR analysis of the expression levels of *Hsp90aa1 and Hsp90ab1* in brain regions and peripheral tissues. Values were calculated relative to vehicle-treated young mice. (**C**) Relative protein level of HSF1, SIRT1 and HSP90 in the heart. Densitometric values were calculated relative to vehicle-treated young mice. (**D**) Representative western blots for HSF1, SIRT1 and HSP90. Data are the mean ± SEM. 3 < *n* < 6/group. *P < 0.05; **P < 0.01; ***P < 0.001. Asterisk indicates the statistically significant difference in the level of induction. ^###^*P* < 0.001; number sign indicates the statistically significant difference between vehicle groups. L.C., loading control; mth, months.

As expected, the mRNA levels of both *Hsf1* and *Sirt1* were not affected by HSP990 treatment (Fig. 6A and Supplementary Material, Fig. S5B). The mRNA levels of *Hsp90ab1*, the gene for the constitutive HSP90 isoform, were higher at 4 h post-dosing when compared with 2 h (Fig. 6B and Supplementary Material, Fig. S5C) in both young and old mice in all tissues analysed, whereas *Hsp90aa1* was only up-regulated in the muscle as had been the case for the 2 h post-dosing. In contrast to *Hspa1a/b*, *Dnajb1* and *Hspb1*, the kinetics of *Hsp90ab1* induction is not known; therefore, it is difficult to interpret the differences between young and old mice as they could be a consequence of a change in the rate of *Hsp90ab1* expression or in the repression of its expression in old mice. We also monitored the protein levels of HSF1, SIRT1 and HSP90 in the heart 4 h after dosing: as for the HS experiment, HSP990 treatment did not induce an alteration in the levels of these HSR regulators (Fig. 6C and D). Interestingly, the post-translational status of HSF1 had begun to revert to the inactive condition, further supporting the idea that HSP gene transcription had been repressed by 4 h post-dosing.

### Stress-induced accumulation of HSPs is comparable in young and old mice

**Figure 7.**
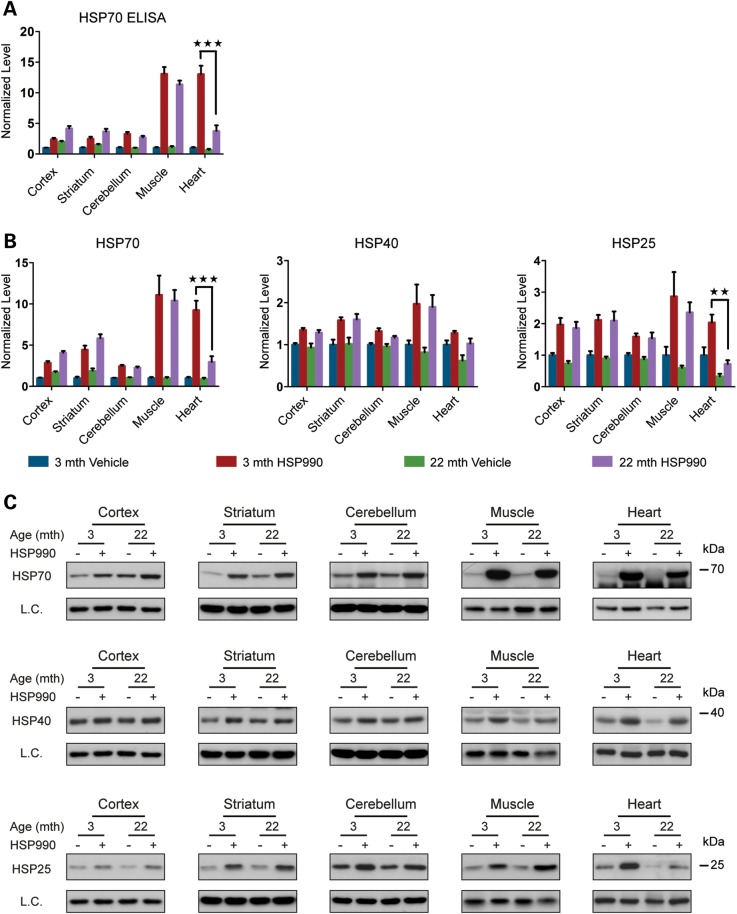
HSP levels 20 h post-treatment with HSP990. Tissues were taken from 3- and 22-month-old mice 20 h after treatment with HSP990 (12 mg/kg) or vehicle. (**A**) ELISA of HSP70 levels in brain regions and peripheral tissues. Values were calculated relative to vehicle-treated young mice. (**B**) Relative protein level of HSP70, HSP40 and HSP25 in brain regions and peripheral tissues. Densitometric values were calculated relative to vehicle-treated young mice. (**C**) Representative western blots used for the quantification in (B). Data are the mean ± SEM. 5 < *n* < 6/group. **P < 0.01; ***P < 0.001. Asterisk indicates the statistically significant difference in the level of induction. Muscle, quadriceps; L.C., loading control; mth, months.

Transcription is the first step in the coordinated process to mount a cellular defence against stress. To determine whether age-related changes in HSP mRNA levels translate into lower levels of HSPs, we treated mice with one acute dose of HSP990 (12 mg/kg) or vehicle and harvested tissues 20 h later. HSP induction follows a much slower kinetics and the fold induction of the proteins is relatively modest when compared with their respective mRNAs (17). Nevertheless, the HSPs are the effectors of the response to stress and therefore it is their induction that is critical for protection against protein misfolding. The availability of an ELISA for HSP70 allowed us to measure the levels of this important chaperone with considerable accuracy; we also measured the levels of HSP70 as well as HSP40 and HSP25 by western blotting. HSP70 levels, as quantified by both ELISA and westerns were highly consistent (Fig. 7A–C and Supplementary Material, Fig. S6A–C) and indicated that post-induction levels in all brain regions and muscle were equivalent between young and old mice and in the range of 2–12-fold. The induction level of HSP40 (1.3–2-fold) and HSP25 (2-fold) did not differ between young and old mice in the tissues listed above (Fig. 7A–C and Supplementary Material, Fig. S6A–C). In contrast, the level of HSP70 and HSP25 induction was decreased in the heart in old when compared with young mice by ∼3-fold, whereas the very modest level of HSP40 up-regulation showed no difference (Fig. 7B and C). Overall, we conclude that HSP induction is altered specifically in the cardiac tissue of old mice and that other tissues, including the brain, are spared from an age-related decline in the HSR.

To determine whether there were age-related effects on HSF1 activation, we monitored the transcription factor by western blotting 20 h after HSP990 treatment. As expected, HSF1 had reverted to its inactive state in HSP990-treated mice in all brain regions and peripheral tissues with no difference being observed between young and old mice (Supplementary Material, Fig. S7A and B). Finally, the 2–4-fold induction of *Hsp90* mRNA that had been detected 2 and 4 h after HSP990 treatment in all tissues was only translated to a significant up-regulation in the amount of HSP90 protein in the muscle and heart (Supplementary Material, Fig. S7A and B), of which the levels in the heart, once again, showed an age-related reduction. Taken together, these data show that the up-regulation of HSPs is only affected by the ageing process in heart tissue.

### The effect of ageing on the expression of the basal levels of HS-related proteins

The preservation of the proteome is a high priority for cells, as this is essential to define cell identity and plasticity and to guarantee that cellular networks function at the higher systems level. It has been documented that metastable, aggregation-prone proteins responsible for neurodegenerative diseases such as Alzheimer's disease, Parkinson's disease and Huntington's disease, severely challenge the proteostasis network, but do not result in the induction of stress response pathways (17,32,33). As such, it may be the capacity of the proteostasis network in basal conditions that dictates cell fate during ageing. To investigate this possibility, we decided to monitor the basal expression levels of the HSR players included in this study, in 3- and 22-month-old mice.

**Figure 8.**
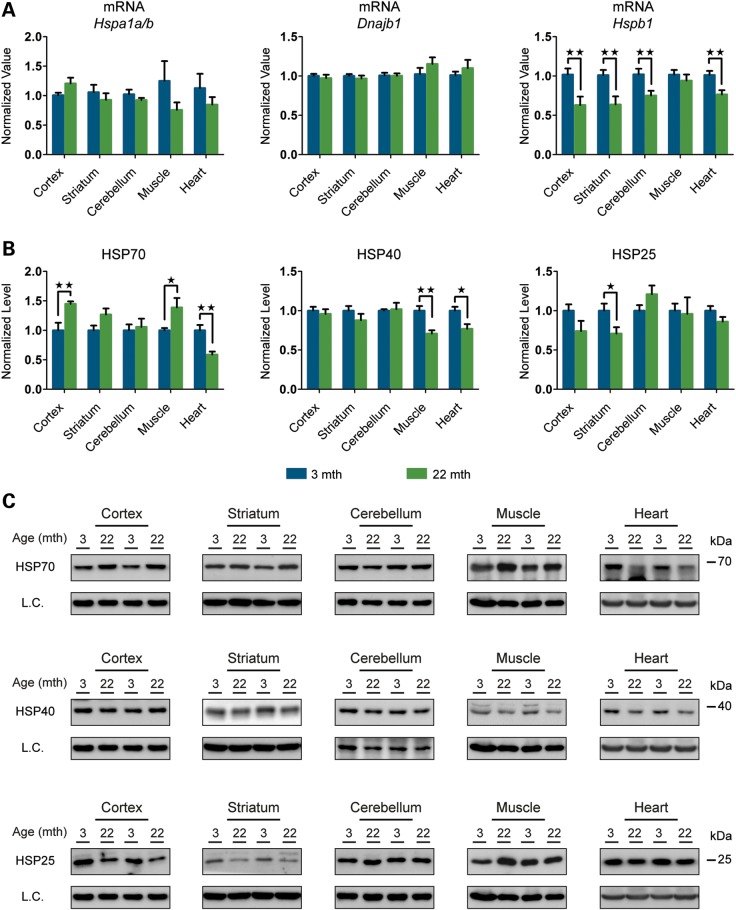
Basal expression levels of the major HSPs in young and old mice. (**A**) qPCR analysis of basal expression levels of *Hspa1a/b*, *Hspb1* and *Dnajb1* in brain regions and peripheral tissues from mice at 22 months of age when compared with 3 months (*n* = 8/age). (**B**) Relative basal levels of HSPs in brain regions and peripheral tissues of mice at 22 months of age when compared with 3 months (*n* = 7/age). (**C**) Representative western blots used for the quantification in (B). Data are the mean ± SEM. *P < 0.05; **P < 0.01. Asterisk indicates the statistically significant difference in the level of induction. Muscle, quadriceps; L.C., loading control; mth, months.

We used both RT–qPCR and western blotting to analyse mRNA and protein levels, respectively. We detected comparable mRNA levels of both *Hspa1a/b* and *Dnajb1* in all brain regions and peripheral tissues, the only exception being the liver where *Hspa1a/b* mRNA was 49% lower in old than in young mice (Fig. 8A and Supplementary Material, Fig. S8A). Interestingly, the mRNA levels of *Hspb1* were highly altered in old mice showing a significant down-regulation of between 25 and 38% in the cortex, striatum, cerebellum, rest of brain and heart (Fig. 8A and Supplementary Material, Fig. S8A). Despite their very stable mRNA levels, HSP70 and HSP40 fluctuated more at the protein level: significant up-regulation of HSP70 levels was detected in old mice in the cortex (45%) and muscle (39%) with the same trend in striatum, whereas the heart and liver showed significant down-regulation of HSP70 of 41 and 49%, respectively (Fig. 8B and C and Supplementary Material, Fig. S8B and C). Similarly, HSP40 was down-regulated in all three peripheral tissues to between 20 and 29%. The down-regulation registered at the mRNA level for *Hspb1* was recapitulated at the protein level in some of the tissues: cortex, striatum and hippocampus (between 26 and 29%), whereas there was a 40% increase in HSP25 in brain stem (Fig. 8B and C and Supplementary Material, Fig. S8B and C).

**Figure 9.**
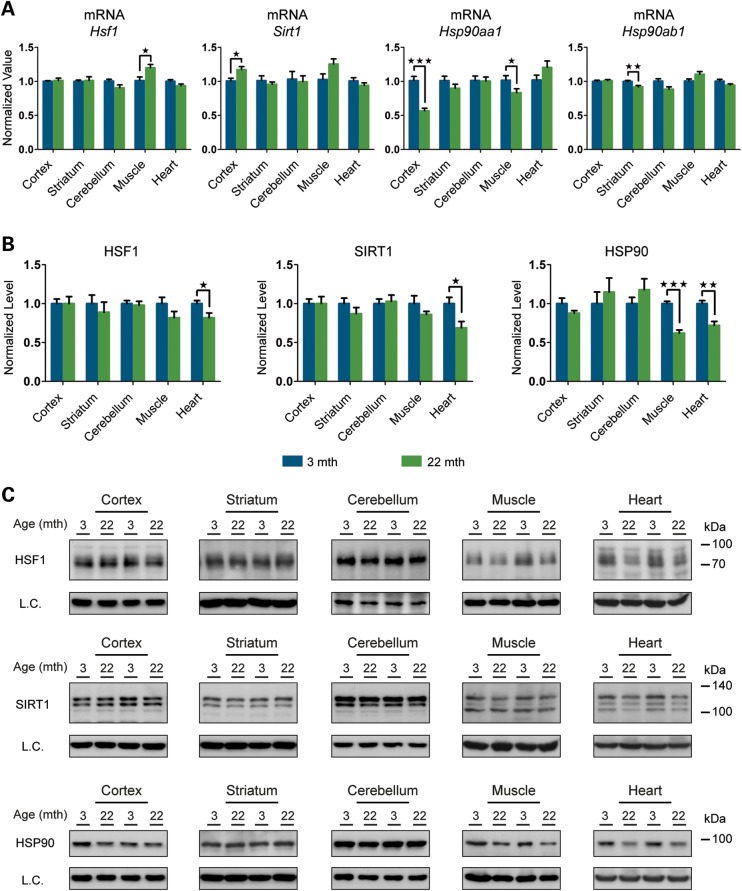
Basal expression levels of the major HSR regulators in young and old mice. (**A**) qPCR analysis of basal expression levels of *Hsf1*, *Sirt1*, *Hsp90aa1* and *Hsp90ab1* in brain regions and peripheral tissues from mice at 22 months of age when compared with 3 months (*n* = 8/age). (**B**) Relative basal protein levels of HS regulators in brain regions and peripheral tissues of mice at 22 months of age when compared with 3 months (*n* = 7/age). (**C**) Representative western blots used for the quantification in (B). Data are the mean ± SEM. *P < 0.05; **P < 0.01; ***P < 0.001. Asterisk indicates the statistically significant difference in the level of induction. Muscle, quadriceps; L.C., loading control; mth, months.

We also monitored the levels of HSF1, SIRT1 and HSP90 as major regulators of the HSR pathway. As observed in all our experiments so far, the variation at the mRNA level for *Hsf1* and *Sirt1* was only minor in all brain regions and peripheral tissues in old when compared with young mice (Fig. 9A and Supplementary Material, Fig. S9A). Protein levels of both HSF1 and SIRT1 were also very stable between the two age groups, and we were able to confirm the down-regulation of these two proteins in the heart of old mice (Fig. 9B and C and Supplementary Material, Fig. S9B and C). The down-regulation of *Hsp90* mRNA in old mouse tissues ranged from a very subtle degree (8–14%) for the constitutive isoform *Hsp90ab1*, to considerable reductions for the inducible isoform *Hsp90aa1* in the cortex (44%), hippocampus (31%) and muscle (19%) (Fig. 9A and Supplementary Material, Fig. S9A). At the protein level, HSP90 was only significantly down-regulated in old mice in the muscle (38%) and heart (28%) (Fig. 9B and C and Supplementary Material, Fig. S9B and C).

In summary, we identified a region-specific up-regulation of HSP70 and down-regulation of HSP25 in the brain. In the periphery, HSP40 and HSP90 are consistently down-regulated in all peripheral tissues. Once again, the heart was the peripheral tissue most affected by the decreased levels of HSR-related proteins in old when compared with young mice.

## DISCUSSION

A decline in proteostasis has been proposed to be a critical mediator of the cell and tissue deterioration that is the characteristic of ageing. There is evidence to suggest that an impairment of cytoprotective stress responses such as the HSR (8), OxSR (34) and ER unfolded protein response (UPR^ER^) (10) leaves cells vulnerable to the environmental and physiological stress that occurs with age, thereby influencing life span and susceptibility to disease. However, the extent to which stress response impairment contributes to age-related cellular decline in mammalian tissues has not been comprehensively investigated.

In the present study, we provide an integrated view of the HSR with insights into the mechanisms behind the tissue-specific regulation of a number of chaperones over time and in multiple tissues. We failed to detect an age-related decline in the HSR in either the brain or the peripheral tissues of old mice but we did find an age-related effect on the regulation of HS-related proteins in the heart. We took advantage of two complementary approaches, HSP90 inhibition and whole body hyperthermia, to investigate the potential of the HSR pathway in mice at 22 months when compared with 3 months of age. The use of a heating pad to raise body temperature is a very effective tool: in an interval of 6–7 min, temperature rises from 36.5° ± 0.2°C to 41.5° ± 0.2°C (∼0.77° ± 0.6°C/min), mimicking the thermal shock that would be experienced by cells and worms in a petri dish. However, exposure to a blood stream at 41.5°C for 15 min was not sufficient to induce the HSR in the brain. This experimental design might be insufficient to cause an HS in brain tissues or it may be that the brain is endowed with a higher threshold for HSR induction. To circumvent this limitation, the use of HSP990 allowed us to study the capacity of the HSR in nine brain regions and peripheral tissues by following the dynamics of the expression of seven genes in parallel.

Interestingly, HS resulted in stronger induction of HSP mRNA than HSP990 treatment in the muscle, whereas in the heart, the levels of all three chaperone genes were between 10- and 20-fold higher after HSP990 treatment than after HS. This finding suggests that the regulation of the HSR may be subtly different between tissues, perhaps owing to cell-type-specific requirements of the proteome and different thresholds or dynamics of gene induction following stress. Irrespective of the stress applied, and consistent with previous data for muscle (12), we were not able to detect an impairment in the HSR in the quadriceps of old mice. HSP990 treatment also revealed no difference in the extent to which the HSR was induced in brain tissue between young and old mice at both the mRNA and the protein level. However, a marked difference in the HSR at the protein level was observed in aged heart tissue.

**Figure 10.**
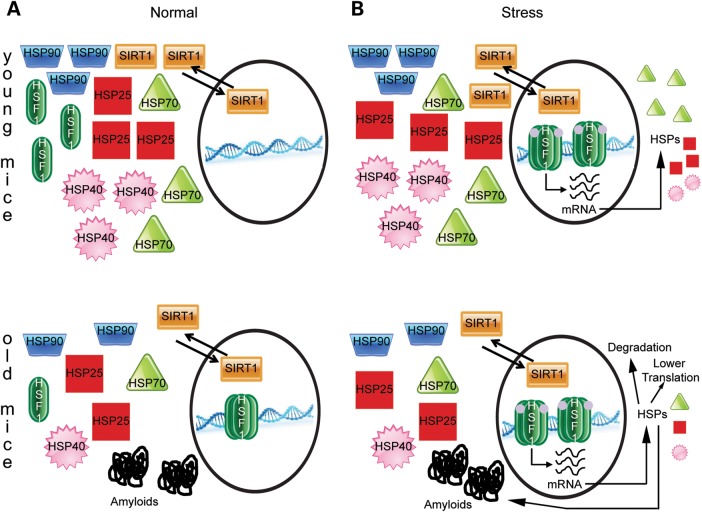
The proposed model for heart compensatory mechanism. (**A**) In normal, non-stressed conditions, HSR-related protein levels are reduced in old hearts and HSF1 is retained at the promoters of HSP genes. (**B**) Under stress conditions, HSF1 becomes hyperphosphorylated and actively promotes the transcription of HSP genes at a rate that is equivalent between young and old mice. Nevertheless, HSP accumulation is lower in old mice which could be a consequence of a slower rate of translation, an increased rate of degradation (perhaps due to a different tissue metabolism arising from compensatory remodelling of the heart) or sequestration of HSPs into amyloid deposits.

Interestingly, there was no age-related difference in the cascade leading to HSF1 activation and consequently DNA-binding capacity in both the brain and the periphery. Previous reports suggest that the failure to up-regulate *Hspa1a/b* with age in the heart and liver is the result of reduced HSF1 DNA-binding activity, as measured by electrophoretic mobility shift assay (11,29,30). However, our ChIP data suggest that in the heart, the reduced levels of HSP70 produced in response to stress are not due to reduced binding of HSF1 to the *Hspa1a/b* promoter, despite the fact that soluble levels of HSF1 are greatly reduced. This suggests that cardiac cells may compensate for reduced HSF1 levels by the constitutive retention of HSF1 at promoters to drive gene expression. This may explain why the difference in protein levels is more pronounced than the difference in mRNA levels and suggests that altered post-transcriptional mechanisms contribute strongly to the HSR alteration in the aged hearts (Fig. 10).

Ageing has a profound effect on the heart and the arterial system and the interaction between the two is balanced to preserve ventricle-atria homeostasis (35). Cardiomyocytes (the contractile cells of the heart) are terminally differentiated cells (36); therefore, quality-control mechanisms for replacing damaged proteins are essential for their health and survival (37). The beneficial effect of stress protein up-regulation in cardiovascular tissue has been widely documented (38), as well as the failure to induce the HSR to a comparable extent in old and young animals (11). In this regard, our data add a further layer of information highlighting some compensatory mechanisms previously unknown. The retention of HSF1 at the promoters of HSP genes in old hearts is an elegant solution to avoid potential delays in activating transcription arising from other age-related deficiencies. This may be why, contrary to previous data (11), at the transcriptional level we were not able to detect an impairment in HSP gene induction in old mice in both the HS experiment and after HSP990 treatment. However, in contrast, the protein profile of HSP990-treated old hearts differed from young ones, with lower levels of HSP70 and HSP25 being present 20 h post-treatment. This could be due to a reduced translational rate in old animals or a different tissue metabolism negatively affecting the half-life of those proteins, as the heart undergoes compensatory remodelling with age often leading to hypertrophy and/or fibrosis.

The observation that the basal levels of HS-related proteins were reduced in the hearts of old mice was an interesting new finding as it does not seem to be a general property of the heart proteome (39). In many tissues, including the heart, cell stress and ageing can cause the accumulation of damaged proteins with highly amyloidogenic potential (40–42). Chaperones may be recruited to misfolded, damaged proteins and become trapped as the proteins oligomerise and form aggregates, decreasing chaperone availability. Intriguingly, the levels of HSF1 and SIRT1 were also reduced in old mice. Although SIRT1 plays a key role in regulating organismal life span and ageing (43), its role in the physiology of heart is controversial and has not yet been entirely clarified (35). Several studies suggest that dependent on the extent of SIRT1 activation in the heart, it can be either beneficial or deleterious, with only a moderate increase positively affecting stress resistance and ageing (35,44). In light of this, reduced levels of SIRT1 in the heart of aged mice may be part of a fine tuning mechanism in an attempt to reach and/or maintain an extremely delicate equilibrium.

To the best of our knowledge, this in-depth, broad range analysis of the HSR in the mouse brain provides the first comprehensive picture of this complex cellular process in mammals. The inability of the brain to deal with chronic proteotoxic stress in neurodegenerative conditions, such as in Alzheimer's disease, Parkinson's disease and Huntington's disease, led to speculations that the HSR is compromised in neurons as a consequence of ageing. Instead, we have shown that in mice, the brain is able to maintain an efficient HSR in old age, suggesting that neurons are not more susceptible to HSR decline or alternatively, that non-neuronal cell types contribute to the seemingly static nature of the HSR with age.

While our data suggest that a decline in the HSR does not occur in all mouse tissues with age, it is possible that other stress response pathways, protein degradation systems or molecular chaperones become impaired with age, thereby causing proteostasis collapse. As such, future studies to assess the effects of aging on other arms of the proteostasis network will be of great interest. Irrespective of this, the development of new intervention strategies to improve the proteostasis capacity of the brain would still be predicted to counteract the accumulation of damaged proteins with beneficial consequences. While surprising, our study has revealed that HS-related proteins are severely compromised in the heart, which, if similar in humans, may be a contributory factor to the high prevalence of senile cardiac amyloid deposits in individuals aged 80 years and over (45–47).

## MATERIALS AND METHODS

### Mouse maintenance

All experimental procedures performed on mice were conducted under a project licence from the Home Office. Wild-type mice were F1 hybrids from a cross between C57BL/6OlaHsd females and CBA/CaOlaHsd males and were purchased from Harlan Olac, Bicester, UK. All animals were subject to a 12-h light/12-h dark cycle and had unlimited access to water and food. Housing conditions and environmental enrichment were as previously described (48). Mouse brain regions and peripheral tissues were snap frozen in liquid nitrogen and stored at −80°C.

### NVP-HSP990 dosing

NVP-HSP990 (23,24) was obtained from Novartis Pharma AG and formulated using a propriety solution. However, 0.2% methyl cellulose can be used as an alternative vehicle. The HSP990 vehicle mixture was briefly sonicated at high frequency in a water bath and mixed thoroughly to form a uniform suspension. Compound or vehicle alone was freshly prepared for each round of treatment and administered to mice by oral gavage, with thorough mixing between dosing to ensure an even suspension. For all experiments, each treatment group contained age- and sex-matched mice. For each specific experiment, young and old mice were weighed and dosed on the same day in the afternoon.

### Heat shock

Animals subjected to HS were anesthetized with isofluorane (Merial Animal Health Ltd) with an oxygen flow of 2 l/min (vaporiser IsoFlo™ Series 5, T.C.V BME; Vet Tech Solutions) and wrapped (whole mouse exclusive of the head) in a heating pad (CWE TC-1000; Linton Instrumentation) set at 41.5°C ± 0.2°C. During HS, core body temperature was maintained at 41.5°C ± 0.2°C for 15 min and was constantly monitored by a rectal probe. Following HS, animals were cooled, revived and returned to their home cage. Control animals were anesthetized and wrapped in a heating pad set at 36.9°C ± 0.2°C. Core body temperature was maintained for 15 min and was constantly monitored by a rectal probe. Four hours after HS, mice were culled by cervical dislocation and tissues were harvested, snap frozen in liquid nitrogen and stored at −80°C.

### Taqman RT–qPCR

RNA extraction, cDNA synthesis, Taqman RT–qPCR and ΔCt analysis were performed as described previously (17,49). The Taqman qPCR assays for *Gapdh*, *Atp5b*, *Rpl13a*, *EiF4a2*, *Ywhaz*, *Sdha*, *Hsp90aa1* and *Hsp90ab1* were purchased from Primer Design. For a list of primers and probes for other assays, see Supplementary Material, Table S1.

### SDS–PAGE and immunoblotting

Frozen mouse brain tissue was homogenized in ice-cold buffer [50 mm Tris–HCl, pH 8.0, 150 mm NaCl, 10% glycerol, 1% Triton X-100, 10 mm ethylenediaminetetraacetic acid (EDTA)] or ELISA buffer (ADI-EKS-700B; Enzo Life Sciences) supplemented with complete protease inhibitors (11697498001; Roche) and phosphatase inhibitors [1 mm sodium orthovanadate (P0758S; New England Biolabs), 50 mm NaF (201154; Sigma), 10 nm okadaic acid (08010; Sigma)]. Protein concentration was determined by BCA assay (23223 and 23224; Thermo Scientific), and 10–20 µg protein was added to 2× Laemmli loading buffer before being subjected to sodium dodecyl sulphate polyacrylamide gel electrophoresis (SDS–PAGE) and western blotting as described previously (50). Membranes were incubated with primary antibody overnight at 4°C in phosphate buffered saline (PBS) with 0.2% Tween (PBST) and 5% non-fat milk for all antibodies except anti-ATP5B and anti-tubulin, which were incubated for 20 min at room temperature. Blots were washed three times for 5 min in PBST, incubated with secondary antibodies in PBST for 1 h at room temperature, washed three times for 5 min in PBST and exposed to ECL according to the manufacturer's recommendations (Amersham). Signal was developed using Amersham hyperfilm and a Xenograph developer. For full details on primary and secondary antibodies, see Supplementary Material, Table S2. ATP5B, β-actin and α-tubulin antibodies were used as loading controls. For full details on the loading controls used for each brain region and peripheral tissues, see Supplementary Material, Table S3.

### Densitometry

Densitometry of western blots was performed using a Bio-Rad GS-800 densitometer. Developed films were scanned, and the average pixel optical density (OD) for each band was measured. The OD of an area devoid of bands was subtracted from the values obtained for bands of interest in order to normalize the OD against background. Relative expression was determined by dividing the normalized OD of bands of interest by the OD of the appropriate loading control for each sample.

### HSP70 ELISA

Sample preparation and ELISA reaction were carried out according to the manufacturer's instructions (ADI-EKS-700B; Enzo Life Sciences).

### Chromatin immunoprecipitation

Frozen brain regions and liver samples were dounce homogenized in ice-cold PBS supplemented with complete protease inhibitors (11697498001; Roche) and phosphatase inhibitors [1 mm sodium orthovanadate (P0758S; New England Biolabs), 50 mm NaF (201154; Sigma), 10 nm okadaic acid (08010; Sigma)]. Muscle and heart samples were reduced to powder using liquid nitrogen and a mortar and pestle, and then resuspended in ice-cold PBS as above. Tissue homogenates were then spun at 500 g for 5 min to remove supernatant (used for IP experiments). Pellets were washed twice with PBS, resuspended in 1 ml of 1% formaldehyde and incubated at 37°C for 15 min. Samples were pelleted and washed twice with PBS before being resuspended in 1 ml ChIP lysis buffer (50 mm Tris–HCl, pH 8.0; 1% SDS; 10 mm EDTA). Samples were then sonicated at high frequency for 15 cycles (30 s on, 30 s off) with a Diogenode Bioruptor sonicator (UCD-200 TO; Diagenode) to yield chromatin fragments of 500–1000 bp in size. After sonication, samples were spun at 10 000 g for 15 min at 4°C, and supernatant was retained. 200–600 µl chromatin (30–50 µg) was incubated with 3 µg of anti-HSF1 antibody (sc-9144 X (H-311 X); Santa Cruz) or rabbit IgG (#2729; Cell Signaling) in dilution buffer (17 mm Tris–HCl, pH 8.0; 167 mm NaCl; 1% Triton-X100; 1.2 mm EDTA) up to 1 ml. Samples were left to rotate overnight at 4°C. 20 µl blocked protein-G Dynabeads (10004D; Life Technologies) were added to each sample and incubated at 4°C for 6 h on a rotating wheel (SB3 rotating wheel; Stuart Instruments). Beads were then washed once in wash buffer 1 (20 mm Tris–HCl, pH 8.0; 150 mm NaCl; 0.1% SDS; 1% Triton X-100; 2 mm EDTA), twice in wash buffer 2 (20 mm Tris–HCl, pH 8.0; 500 mm NaCl; 0.1% SDS; 1% Triton X-100; 2 mm EDTA) and twice in wash buffer 3 (10 mm Tris–HCl, pH 8.0; 250 mm LiCl; 1% NP-40; 1% Na deoxycholate; 1 mm EDTA) before being resuspended in 120 µl elution buffer (50 mm Tris–HCl pH 8.0, 10 mm EDTA and 1% SDS). Beads were incubated at 65°C overnight. Chromatin was purified using the iPure kit (AL-100-0100; Diagenode) as per the manufacturer's instructions. 10% input chromatin was also de-cross-linked and purified as described above. SYBR green qPCR was then carried out on pulldown and input samples, and the ΔCt method was used to determine relative amounts of DNA. Cycling parameters were as follows: 3 min at 95°C, (10 s at 95°C, 30 s at 60°C) for 45 cycles, followed by a melting curve from 59°C to 95°C with a 0.5°C increment every 10 s.

### Immunoprecipitation

Tissues were dounce homogenized in ice-cold PBS supplemen-ted with complete protease inhibitors (11697498001; Roche) and phosphatase inhibitors [1 mm sodium orthovanadate (P0758S; New England Biolabs), 50 mm NaF (201154; Sigma), 10 nm okadaic acid (08010; Sigma)]. Lysates were used immediately and never frozen. Samples were spun at 13 000 g at 4°C for 15 min, and the supernatants transferred to a fresh tube. Protein concentrations were determined using a BCA-protein assay (23 223 and 23 224; Thermo Scientific). 0.2–0.5 mg protein lysate was incubated with 3 µg anti-HSF1 antibody (SPA-901; Stressgen) or normal rabbit IgG (#2729; Cell Signaling) for 4 h at 4°C on a rotating wheel (SB3 rotating wheel; Stuart Instruments). Protein G-coupled Dynabeads (10004D; Life Technologies) were added to each IP and incubated at 4°C for 45 min on a rotating wheel. Following immunoprecipitation, the protein G-coupled Dynabeads were briefly spun and put on a magnetic rack, washed in 4% Triton X-100/PBS (3×) and finally resuspended in 4× loading buffer (NP0007; Life Technologies) with a reducing agent (NP0004; Life Technologies). Immunoprecipitated complexes were eluted by denaturation at 95°C for 10 min and run on a 4–12% precasted gel (WG1402BOX; Life Technologies) according to the manufacturer's instructions.

### Statistics

All data were screened for statistical outliers using Grubb's Test (GraphPad Software, CA, USA). For data where four groups were analysed, such as the HSP990 dosing and HS experiment, these were analysed using a two-way ANOVA with treatment and age as between-subject factors. Tukey's *post hoc* analysis was applied for multiple comparisons. Main effects of statistical analyses of interest were those that related to (i) comparisons of the vehicle groups between young and old mice and (ii) the level of chaperone induction in either young or old mice. For tests with only two groups, such as the basal level analysis, an unpaired *t*-test was used. Statistical analyses were calculated using SPSS Statistics Ver.21 (IBM, Portsmouth, UK). *P*-values of <0.05 were considered significant. Graphs were constructed using Prism Ver.5.0b (GraphPad Software).

## SUPPLEMENTARY MATERIAL

Supplementary Material is available at *HMG* online.

## FUNDING

This work was funded by the Medical Research Council (G0801314), the CHDI Foundation and the Leslie Gehry Brenner prize from the Hereditary Disease Foundation to G.B. Funding to pay the Open Access publication charges for this article was provided by the Medical Research Council.

## Supplementary Material

Supplementary Data
