# Supplementary material for: Contesting the dogma of an age-related heat shock response impairment: implications for cardiac-specific age-related disorders
Source: Hum Mol Genet. 2014 Feb 19;23(14):3641–56. doi: 10.1093/hmg/ddu073 (PMC4065144; doi:10.1093/hmg/ddu073)
Supplement: Supplementary Data [file supp_23_14_3641__index.html]

Contesting the dogma of an age-related heat shock response impairment; implications for cardiac-specific age-related disorders — Contesting the dogma of an age-related heat shock response impairment: implications for cardiac-specific age-related disorders — Contesting the dogma of an age-related heat shock response impairment: implications for cardiac-specific age-related disorders — Supplementary Data 

# Contesting the dogma of an age-related heat shock response impairment: implications for cardiac-specific age-related disorders

## Supplementary Data

Supplementary Data

**Files in this Data Supplement:**

- Supplementary Data - Pdf file
