# Supplementary material for: Contesting the dogma of an age-related heat shock response impairment: implications for cardiac-specific age-related disorders
Source: Hum Mol Genet. 2014 Feb 19;23(14):3641–56. doi: 10.1093/hmg/ddu073 (PMC4065144; doi:10.1093/hmg/ddu073)
Supplement: Supplementary Data [file supp_ddu073_ddu073supp.pdf]

SUPPLEMENTARY MATERIAL

Contesting the dogma of an age-related heat shock response impairment; implications for cardiac-specific age-related disorders

Alisia Carnemolla[1], John P. Labbadia[1], Hayley Lazell[1], Andreas Neueder[1], Saliha Moussaoui[2,3] and Gillian P Bates[1]*

[1]Dept. Medical and Molecular Genetics, King's College London, London SE1 9RT, UK

[2]Novartis Institute for Biomedical Research, Neuroscience Discovery, CH-4002, Basel, Switzerland.

[3]Current address: Rhenovia Pharma, 20C rue Chemnitz, F-68200 Mulhouse, France

SUPPLEMENTARY FIGURES

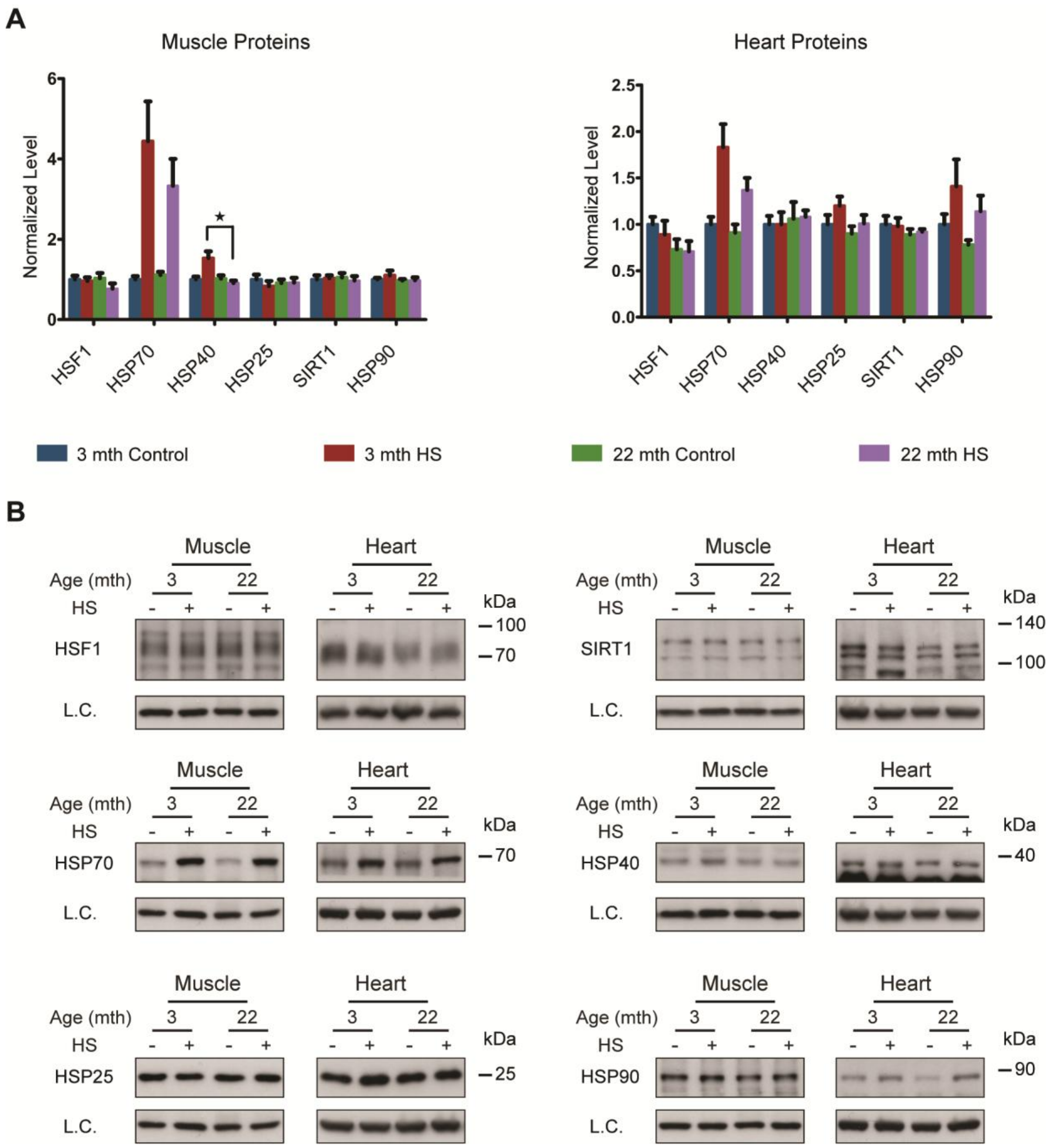

Supplementary Figure S1. Effect of heat shock on HSP induction in young and old mice. Tissues were taken from 3 month and 22 month old mice 4 hours after heat shock (15 minutes at 41.5°C). Controls were maintained at 36.9°C during this period. (A) Relative protein level of HSF1, HSP70, HSP40, HSP25, SIRT1 and HSP90 in muscle and heart. Densitometric values were calculated relative to control young mice. (B) Representative western blots used for the quantification in A. Data are mean ± SEM. 4< n <6 / group. *p < 0.05. *indicates statistically significant difference in the level of induction. L.C. = loading control; mth = months.

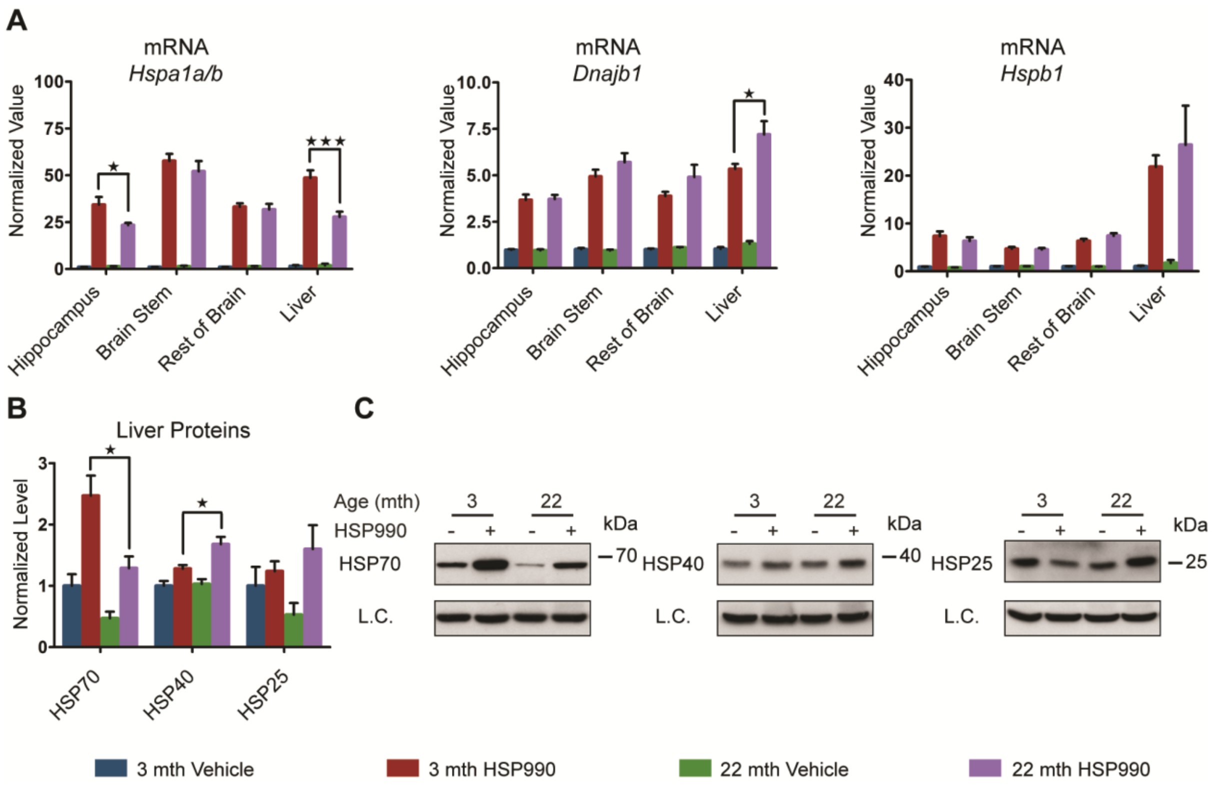

Supplementary Figure S2. HSP induction is not impaired in old mice. Tissues were taken from 3 month and 22 month old mice 2 hours after treatment with HSP990 (12 mg/kg) or vehicle. (A) qPCR analysis of the expression levels of *Hspa1a/b*, *Dnajb1* and *Hspb1* in brain regions and liver. Values were calculated relative to vehicle-treated young mice. (B) Relative protein level of HSP70, HSP40 and HSP25 in liver. Densitometric values were calculated relative to vehicle-treated young mice. (C) Representative western blots used for the quantification in B. Data are mean ± SEM. 4< n <8 / group. *p < 0.05; ***p < 0.001. *indicates statistically significant difference in the level of induction. L.C. = loading control; mth = months.

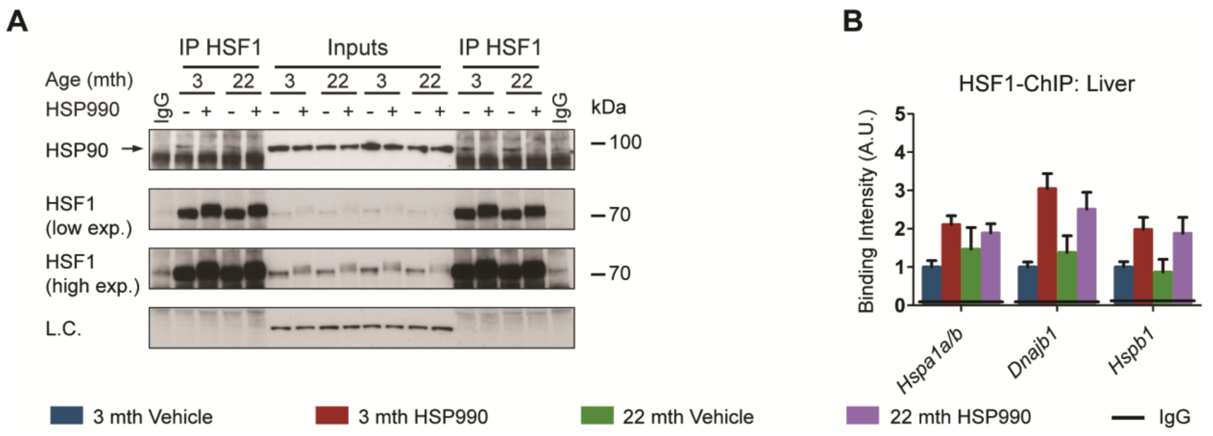

Supplementary Figure S3. HSF1 activation and DNA binding capacity are not negatively affected by ageing. Tissues were taken from 3 month and 22 month old mice 2 hours after treatment with HSP990 (12 mg/kg) or vehicle. (A) Western blots of HSF1 and HSP90 after HSF1 IP from cortical lysates. (B) Levels of HSF1 bound to HS promoters in liver as determined by ChIP. Solid line represents the IgG control value. Data are mean ± SEM. 4< n <8 / group. *indicates statistically significant difference in the level of induction. L.C. = loading control; mth = months. A.U. = arbitrary units.

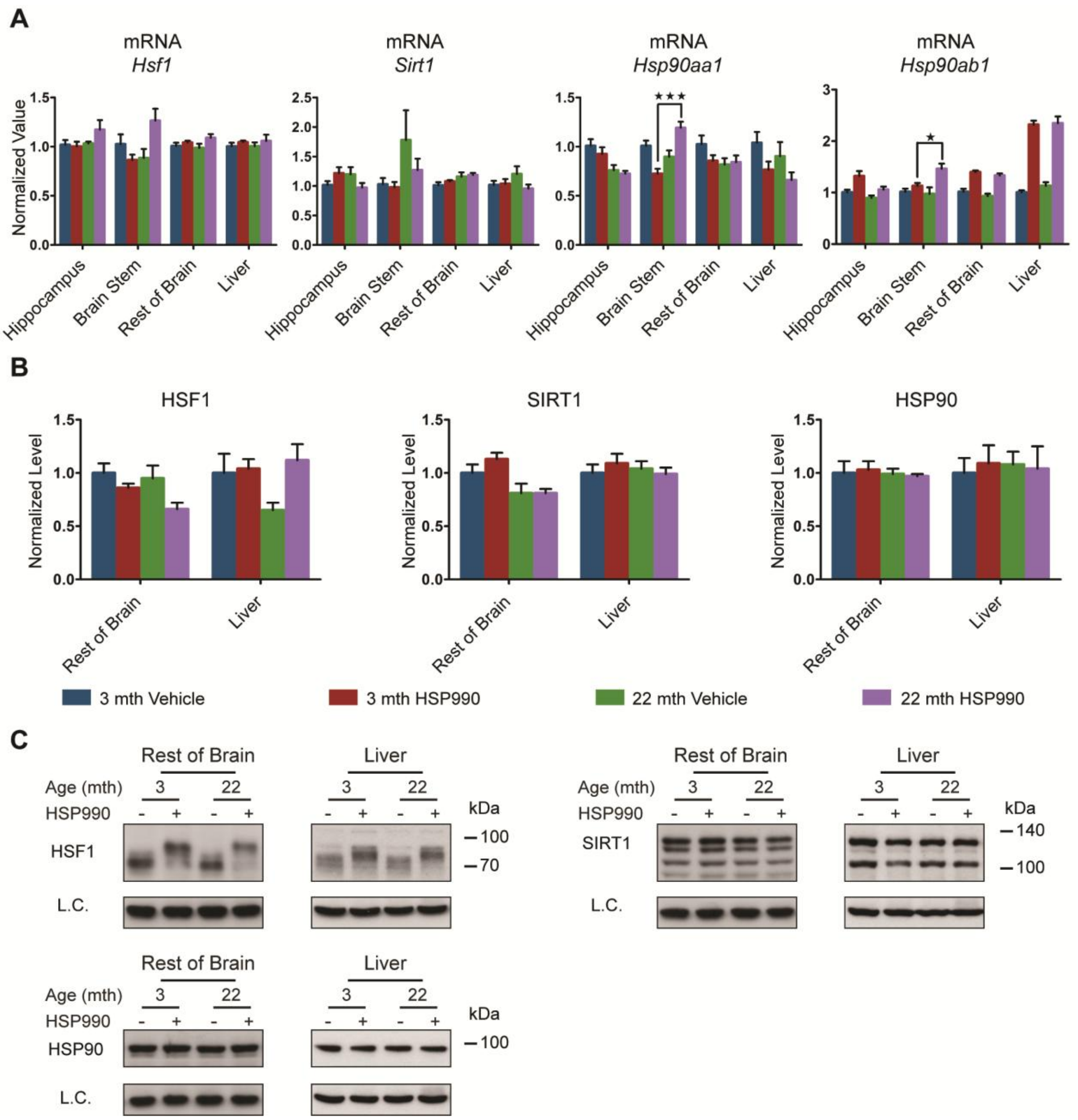

Supplementary Figure S4. Analysis of HSR regulatory proteins. Tissues were taken from 3 month and 22 month old mice 2 hours after treatment with HSP990 (12 mg/kg) or vehicle. (A) qPCR analysis of the expression levels of *Hsf1*, *Sirt1*, *Hsp90aa1* and *Hsp90ab1* in brain regions and liver. Values were calculated relative to vehicle-treated young mice. (B) Relative protein level of HSF1, SIRT1 and HSP90 in rest of brain and liver. Densitometric values were calculated relative to vehicle-treated young mice. (C) Representative western blots for HSF1, SIRT1 and HSP90 in rest of brain and liver. Data are mean ± SEM. 4< n <8 / group. *p < 0.05; ***p < 0.001. *indicates statistically significant difference in the level of induction. L.C. = loading control; mth = months.

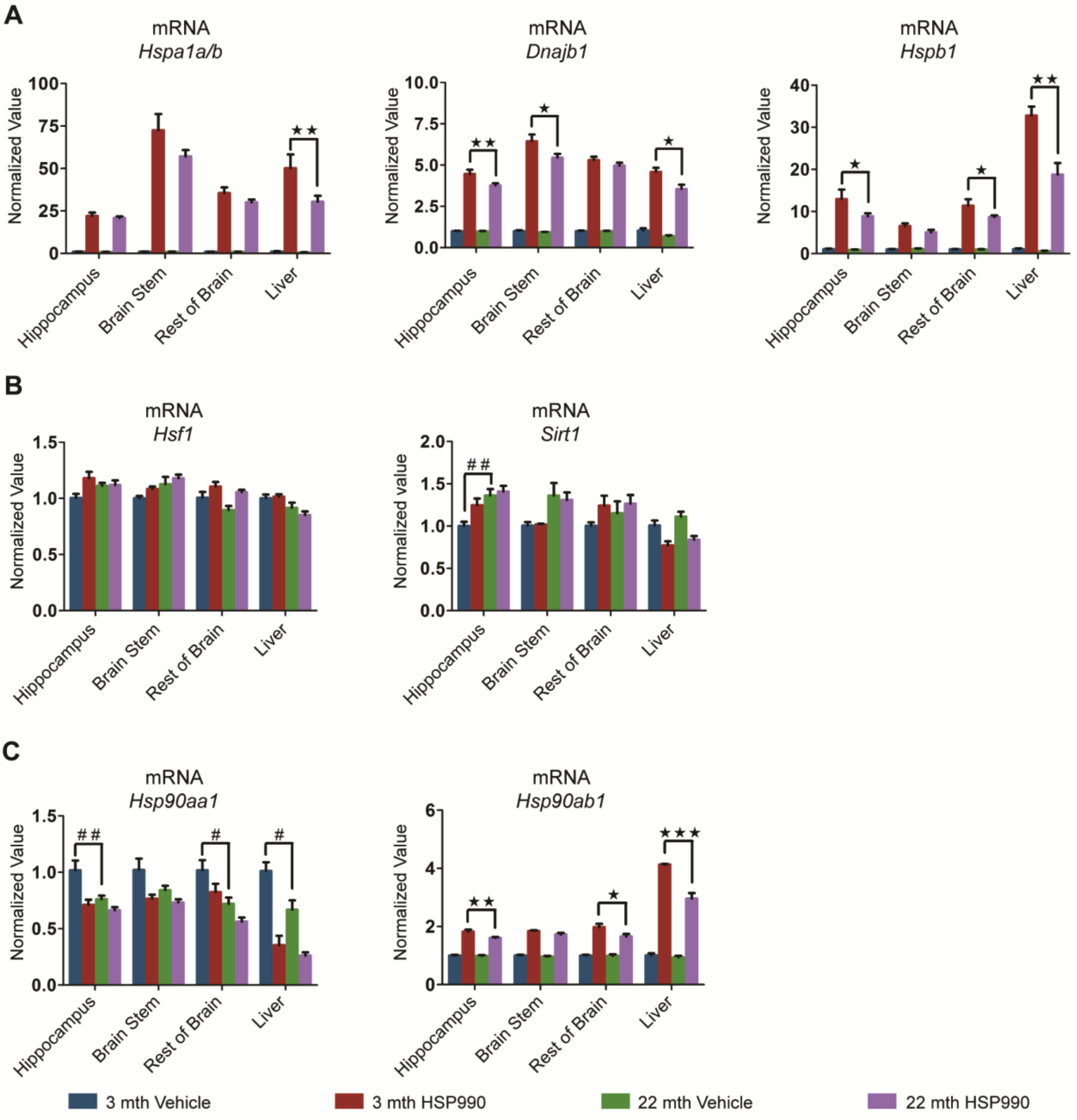

Supplementary Figure S5. Analysis of the dynamics of HSP induction. Tissues were taken from 3 month and 22 month old mice 4 hours after treatment with HSP990 (12 mg/kg) or vehicle. (A) qPCR analysis of the expression levels of *Hspa1a/b, Dnajb1* and *Hspb1* in brain regions and liver. (B) qPCR analysis of the expression levels of *Hsf1* and *Sirt1* in brain regions and liver. (C) qPCR analysis of the expression levels of *Hsp90aa1 and Hsp90ab1* in brain regions and liver. Values were calculated relative to vehicle-treated young mice. Data are mean ± SEM. 4< n <8 / group. *$p < 0.05$; **$p < 0.01$; ***$p < 0.001$. *indicates statistically significant difference in the level of induction. $^{\#}p < 0.05$, $^{\#\#}p < 0.01$: $^{\#}$indicates statistically significant difference between vehicle groups. mth = months.

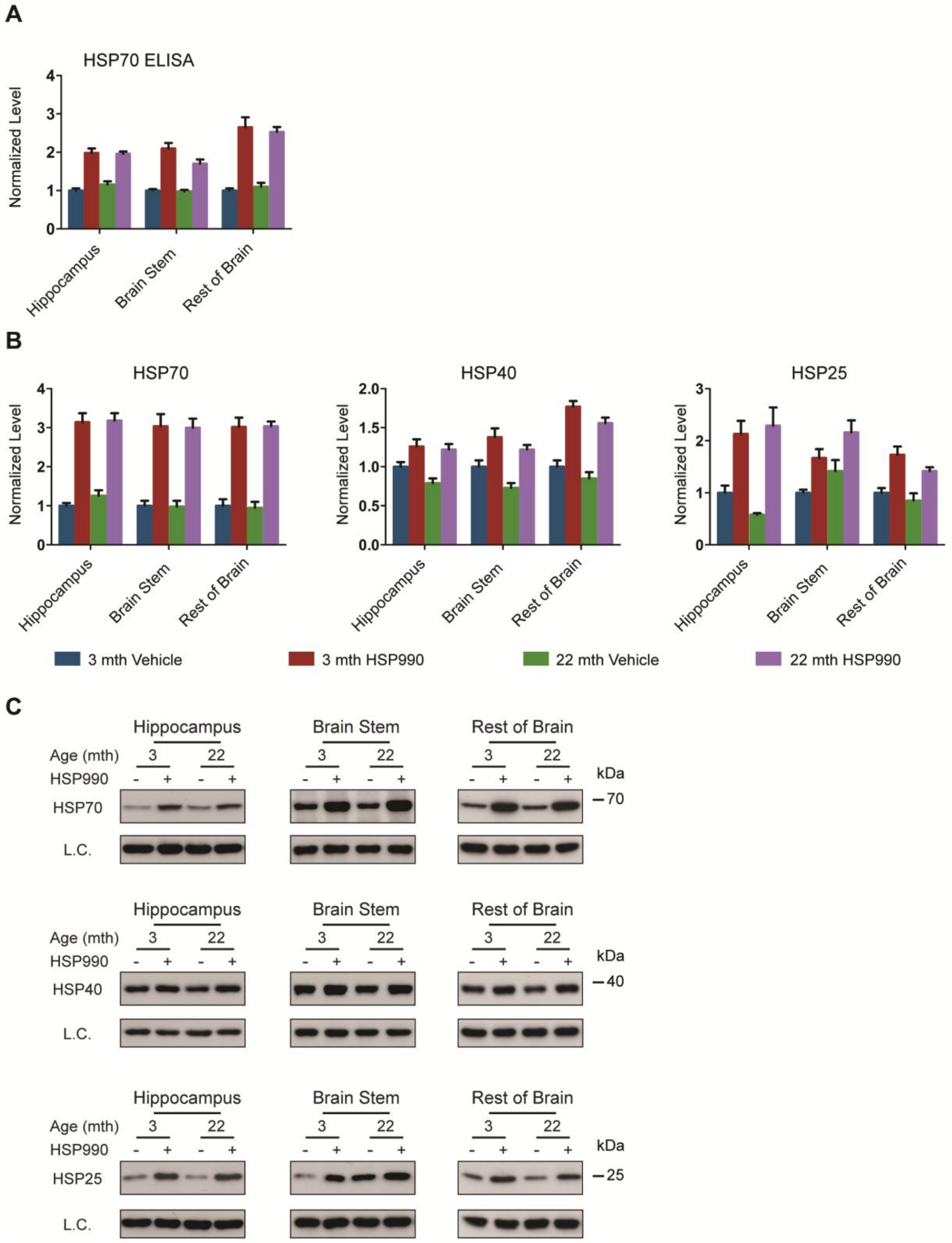

Supplementary Figure S6. HSP levels 20 hours post treatment with HSP990. Tissues were taken from 3 month and 22 month old mice 20 hours after treatment with HSP990 (12 mg/kg) or vehicle. (A) ELISA of HSP70 levels in brain regions. Values were calculated relative to vehicle-treated young mice. (B) Relative protein level of HSP70, HSP40 and HSP25 in brain regions. Densitometric values were calculated relative to vehicle-treated young mice. (C) Representative western blots used for the quantification in B. Data are mean ± SEM. 5< n <6 / group. L.C. = loading control; mth = months.

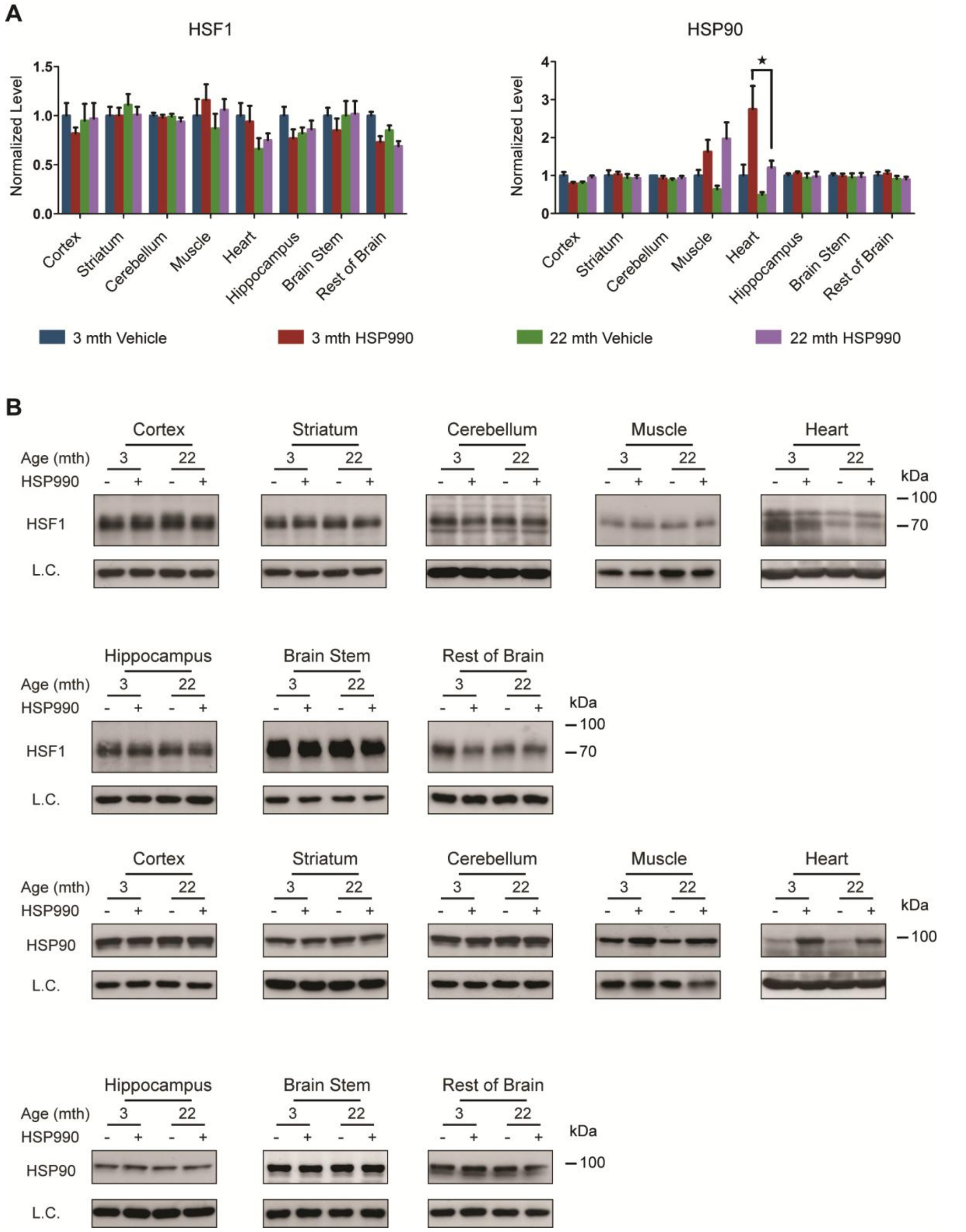

Supplementary Figure S7. HSF1 and HSP90 levels 20 hours post treatment with HSP990. Tissues were taken from 3 month and 22 month old mice 20 hours after treatment with HSP990 (12 mg/kg) or vehicle. (A) Relative protein level of HSF1 and HSP90 in brain regions and peripheral tissues. Densitometric values were calculated relative to vehicle-treated young mice. (B) Representative western blots used for the quantification in A. Data are mean ± SEM. 5< n <6 / group. $*p < 0.05$. *indicates statistically significant difference in the level of induction. L.C. = loading control; mth = months.

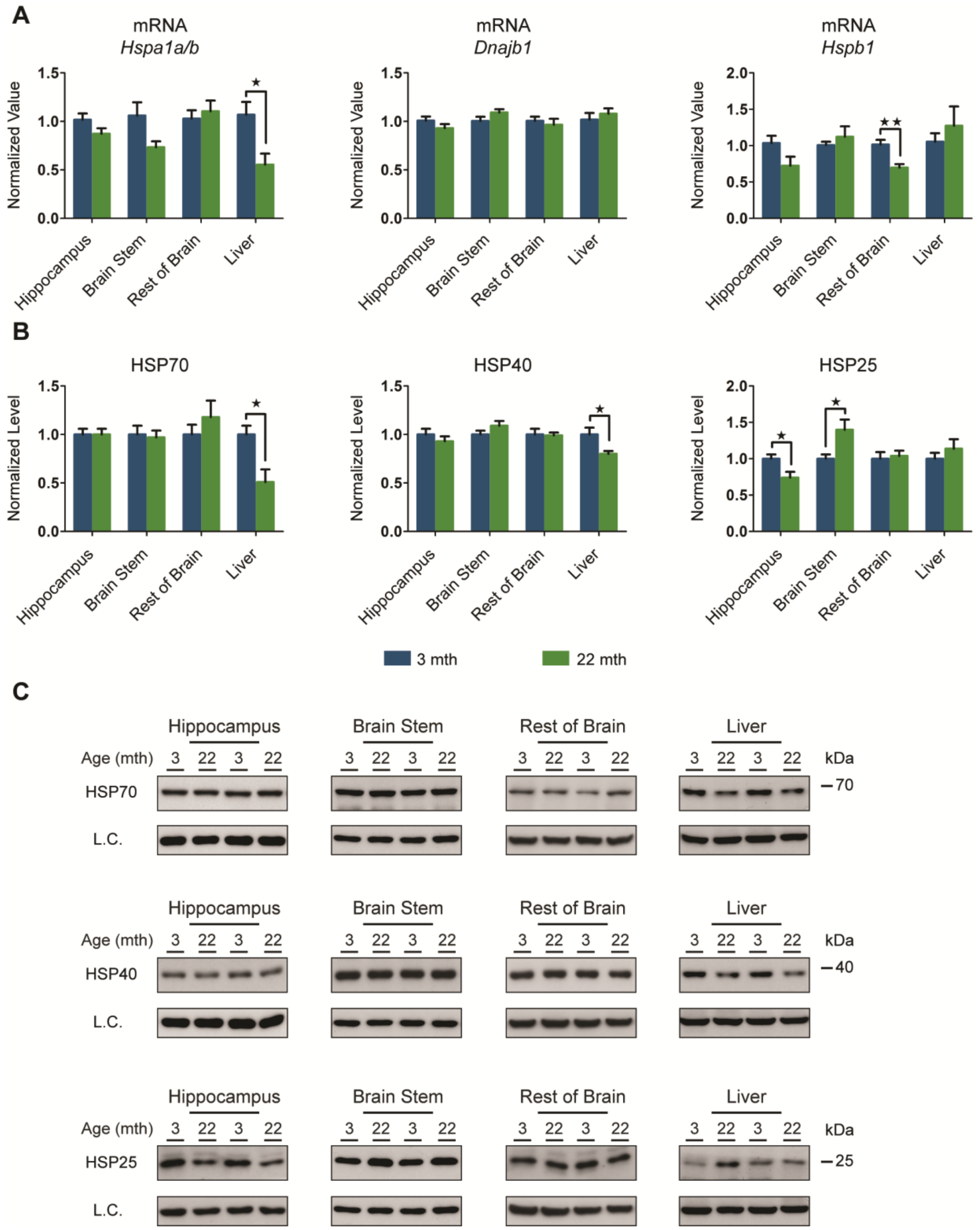

Figure S8. Basal expression levels of the major HSPs in young and old mice. (A) qPCR analysis of basal expression levels of *Hspa1a/b*, *Hspb1* and *Dnajb1* in brain regions and liver from mice at 22 months of age as compared to 3 months (n = 8 / age). (B) Relative basal levels of heat shock proteins in brain regions and liver of mice at 22 months of age as compared to 3 months (n = 7 / age). (C) Representative western blots used for the quantification in B. Data are mean ± SEM. *$p < 0.05$; **$p < 0.01$. *indicates statistically significant difference in the level of induction. L.C.= loading control; mth = months.

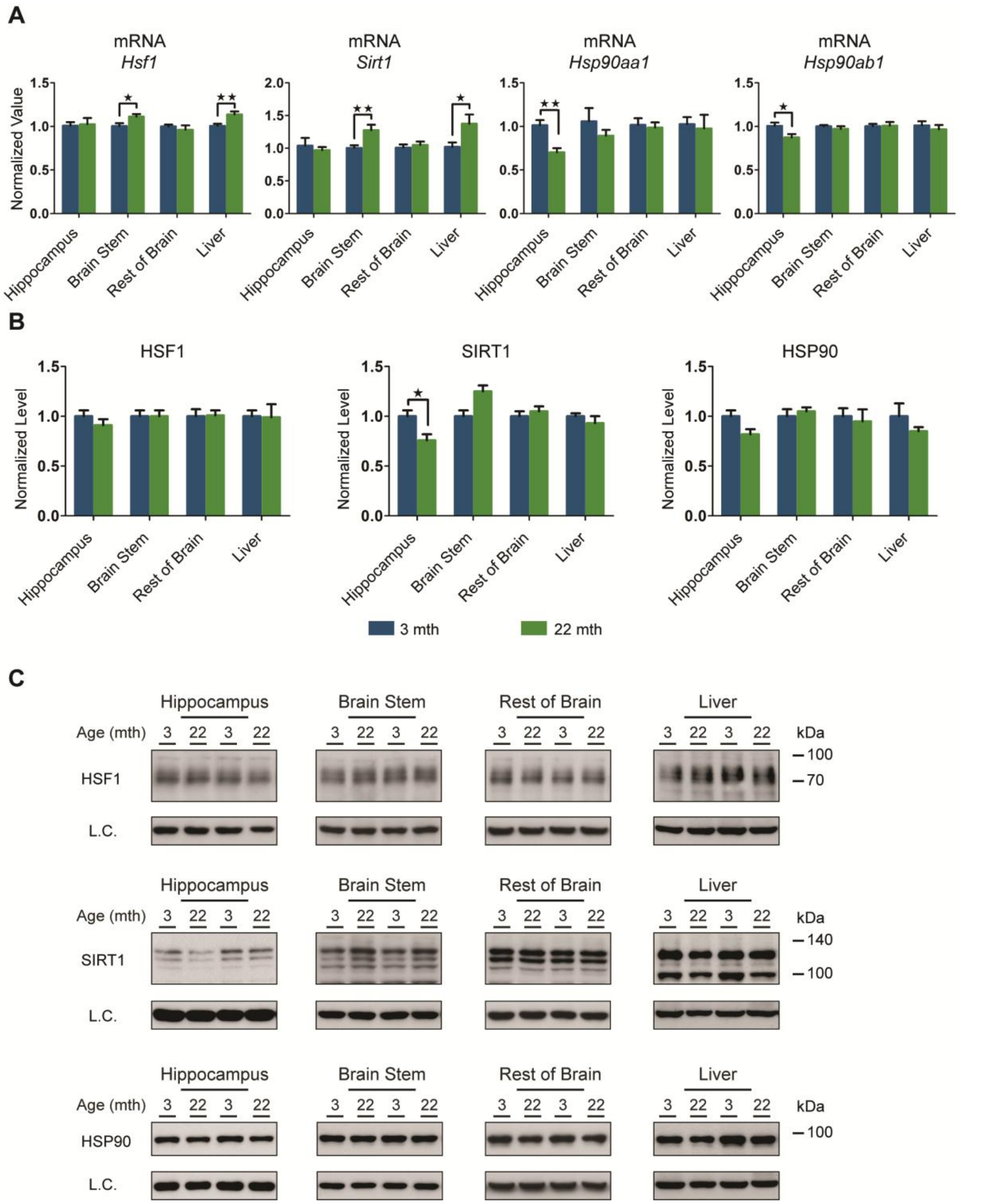

Figure S9. Basal expression levels of the major HSR regulators in young and old mice. (A) qPCR analysis of basal expression levels of *Hsf1*, *Sirt1*, *Hsp90aa1* and *Hsp90ab1* in brain regions and liver from mice at 22 months of age as compared to 3 months (n= 8 / age). (B) Relative basal protein levels of heat shock regulators in brain regions and liver of mice at 22 months of age as compared to 3 months (n = 7 / age). (C) Representative western blots used for the quantification in B. Data are mean ± SEM. *p < 0.05; **p < 0.01. *indicates statistically significant difference in the level of induction. L.C.= loading control; mth = μοντησ.

Supplementary Table S1: RT-qPCR primers and probes

Name	Application	Forward	Reverse	Probe
Hsf1 ex1-2	Taqman	CGAGTGGGAACAGCTTCCA	ACTTGGGCAGCACCTCCTT	TTTGACCAGGGCCAGTT
Hspa1a/b	Taqman	GGTGGTGCAGTCCGACATG	TTGGGCTTGTCGCCGT	CACTGGCCCTTCCAGGTGGTGAA
Dnajb1	Taqman	CCCCATGCCATGTTTGCT	GCGCTGCCCAAAAAAAGG	TCTTCGGTGGCAGAAACCCCTTTGA
Hspb1	Taqman	CACTGGCAAGCACGAAGAAAG	GCGTGTATTTCCGGGTGAAG	ACCGAGAGATGTAGCCATGTTCGTCCTG
Sirt1	Taqman	TGTTGGTTGACTTCATCTTCCTT	TCCAATGGCTTTTGAAAACTTTA	TTCATTTGTATGATACATTCGTATGTATG
Hspa1a/b Promoter	SYBR Green	GCCGGTGAAGACTCCTTAAA	GCTTGTCTCTGGATGGAACC	N/A
Dnajb1 Promoter	SYBR Green	CGCCGGACGGGTATATAGAG	GGCCCAGCGTCTGATAGTAG	N/A
Hspb1 Promoter	SYBR Green	GGCTCCAGTCCGGCACTTCT	GGCGCTCGGTCATGTTCTTG	N/A

Table S2: Antibodies: source, application and working dilutions

Antibody	Catalogue number	Source	Dilution/ Amount	Application
HSF1	Ab81279	Abcam	1:1000	WB
HSF1	ADI-SPA	Stressgen	3 µg	Ip
HSF1	H-311X	Santa Cruz	3 µg	ChIp
SIRT1	ab12193	Abcam	1:1000	WB
HSP90	ADI-SPA-835	Stressgen	1:2000	WB
HSP70	ADI-SPA-810	Stressgen	1:1000	WB
HSP40	ADI-SPA-400	Stressgen	1:5000	WB
HSP25	ADI-SPA-801	Stressgen	1:1000	WB
β-actin	sc-47778	Santa Cruz	1:5000	WB
ATP5b	ab14730	Abcam	1:15000	WB
α-tubulin	T9026	Sigma	1:30000	WB
anti-mouse	P0260	Dako	1:5000	WB
anti-rat	P0450	Dako	1:5000	WB
anti-rabbit	32460	Pierce	1:15000	WB

Key: WB = western Blotting; IP = immunoprecipitation; ChIP = chromatin immunoprecipitation

Table S3: loading controls used for each tissues

Brain region/Tissue	Loading Control
Cortex	β-actin
Striatum	ATP5B
Cerebellum	β-actin
Hippocampus	β-actin
Brain Stem	β-actin
Rest of Brain	β-actin
Muscle (quadriceps)	ATP5B/α-TUBULIN
Heart	ATP5B
Liver	ATP5B
